# Summary of the Current Status of DNA Vaccination for Alzheimer Disease

**DOI:** 10.3390/vaccines11111706

**Published:** 2023-11-10

**Authors:** Caterina Vicidomini, Nicola Borbone, Valentina Roviello, Giovanni N. Roviello, Giorgia Oliviero

**Affiliations:** 1 Institute of Biostructures and Bioimaging, Italian National Council for Research (IBB-CNR), Area di Ricerca Site and Headquarters, Via Pietro Castellino 111, 80131 Naples, Italy; 2 Department of Pharmacy, University of Naples Federico II, Via Domenico Montesano 49, 80131 Naples, Italy; 3 Center for Life Sciences and Technologies (CESTEV), University of Naples Federico II, Via Tommaso De Amicis 95, 80131 Naples, Italy; 4 Department of Molecular Medicine and Medical Biotechnologies, Via Sergio Pansini 5, 80131 Naples, Italy

**Keywords:** DNA vaccines, RNA vaccines, neuropathy, amyloid beta, Alzheimer disease

## Abstract

Alzheimer disease (AD) is one of the most common and disabling neuropathies in the ever-growing aged population around the world, that especially affects Western countries. We are in urgent need of finding an effective therapy but also a valid prophylactic means of preventing AD. There is a growing attention currently paid to DNA vaccination, a technology particularly used during the COVID-19 era, which can be used also to potentially prevent or modify the course of neurological diseases, including AD. This paper aims to discuss the main features and hurdles encountered in the immunization and therapy against AD using DNA vaccine technology. Ultimately, this work aims to effectively promote the efforts in research for the development of safe and effective DNA and RNA vaccines for AD.

## 1. Introduction

Neurological diseases constitute one of the major health issues worldwide. More than 250 million cases of neurological disorders are reported yearly worldwide, with the neurological disorders being the second leading cause of death in developed countries [1]. In general, lifestyle changes are the most common cause of the significantly increased risk of chronic diseases, which places significant financial and health burdens on global society. Chronic dysfunction of nerve cells, both central and peripheral, characteristic of neurological disorders, can lead to irreversible cell damage and death. Unfortunately, the molecular causes of neuropathy are not fully understood. For example, Alzheimer disease (AD) is characterized by the presence of amyloid beta-containing plaques and tau-containing neurofibrillary tangles [2,3,4]. AD is an often hereditary, sporadic neuropathy responsible for amnestic cognitive deficits in the prototypical variant as well as non-amnestic cognitive deficits in other less-common forms. It causes acquired cognitive impairments in mid-to-late life, but its clinical impact on patients is modified by other cerebrovascular and neurodegenerative conditions [5,6]. In this context, researchers are still struggling to find biomolecular targets for therapies that have the potential to alter significantly the clinical course of individuals affected by AD.

### Vaccines and Neurological Diseases

Vaccines prevent millions of diseases and save numerous lives every year. Thanks to the widespread use of vaccines, the smallpox virus has been completely eliminated and the incidence of polio, measles, and other childhood diseases has decreased significantly worldwide [7]. Conventional prophylactic approaches, such as vaccines based on live attenuated and inactivated pathogens as well as subunit vaccines, provide long-term protection against diseases of varying virulence [8]. Despite this encouraging achievement, major obstacles still remain in developing vaccines against the various pathogens responsible for infective diseases or other pathologic conditions, especially those that show a high tendency to evade the adaptive immune response of the human body [9]. Not less importantly, for most vaccines developed against emerging diseases, one main impairment resides in the efficacy of conventional strategies and in the necessity of a large-scale deployment as well as of a more rapid development. Finally, conventional vaccination methods may not be applicable to the so-called non-communicable diseases [10], such as cancer or neurological diseases. Therefore, we urgently need more robust and flexible vaccination approaches, and in this context nucleic acid-based vaccines are a powerful alternative to conventional vaccines thanks to some favorable properties, including their fast development, high efficacy, safer administration, and, not less importantly, low-cost manufacturing. Nonetheless, the application of DNA and mRNA vaccines until recently has been hampered by some obstacles such as the instability and inefficient in vivo delivery of their nucleic acid constituents. Recently, technological advances took place during the COVID-19 pandemic that have largely overcome these problems, and several nucleic acid-based vaccine platforms against certain types of cancer [11,12,13], antimicrobial resistance, and infectious diseases have shown encouraging results in both animal models and in patients [14,15,16]. In 1999, Schenk et al. reported that amyloid beta (Aβ) deposits in AD animal models could be significantly reduced by active vaccination using Aβ peptide [17]. However, a certain toxicity of amyloid vaccination was reported in the scientific literature [18]. Hence, it is important to discover new prophylactic platforms that combine a satisfactory effectiveness with an acceptable level of safety for patients. Interestingly, nucleic acid-based vaccines and especially DNA vaccines are being explored in the fight against Alzheimer disease and show promising features, as we discuss in the next section.

## 2. Alzheimer Disease

Alzheimer disease is the main cause of dementia in older adults and is characterized by progressive neurodegeneration [19]. In 1907, Alois Alzheimer described the case of a 51-year-old patient who suffered from relatively rapid memory loss, as well as mental disorders, who died four years later [20]. Although many progressive and life-threatening neurological disorders had been described at that time, including senile dementia, the early age of onset in the woman examined in 1907 by Alzheimer and the observation of peculiar new features, including the neurofibrillary tangles, associable only to a new pathologic condition, made this neuropathy unique in the eyes of its first discoverers. Whether AD should be regarded as a nosological entity different from the other neurological diseases or not, and why the psychiatrist Emil Kraepelin [21] was inclined to consider AD a new pathological condition continue to be a matter of debate. However, AD today is considered a progressive neurodegenerative condition accompanied by a characteristic pathology [20]. Alzheimer disease was classified into two clinical conditions based on the age of onset of the disease. Since AD was initially described in a relatively young woman, ‘Alzheimer disease’ was the term used for a type of pre-senile dementia affecting people under 65, while a similar condition in over-65 patients was usually referred to as senile dementia or ‘Alzheimer type’ disease, according to pioneering studies on AD [20]. Nowadays, AD is widely recognized as a unique entity whose prevalence increases significantly after age 65. AD must be distinguished from other causes of dementia, such as Lewy body dementia, vascular dementia, frontotemporal dementia, Parkinson’s disease with dementia, and reversible dementia [22,23].

### Diagnosis of Alzheimer Disease

**Figure 1:**
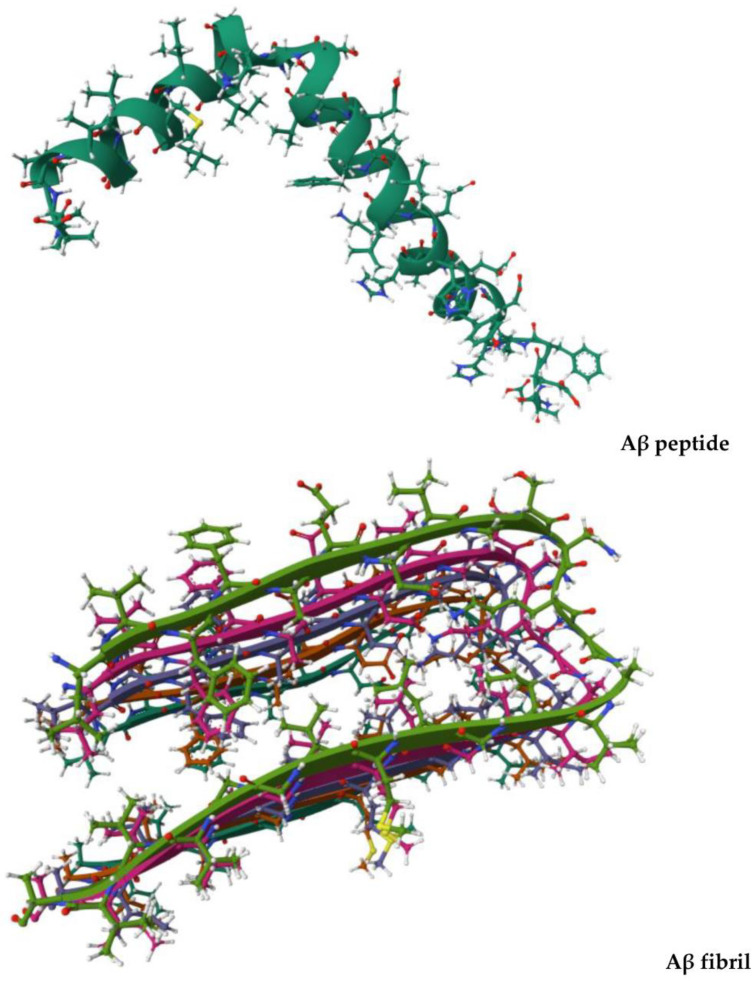
Three-dimensional views of Aβ monomer (Aβ_1–42_, PDB ID: 1IYT, up) and Aβ fibril (Aβ_1–42_ fibrils, PDB ID: 2BEG, down), whose self-assembly leads to amyloid plaques. The reported structures are freely available at the links https://www.rcsb.org/3d-view/1IYT/0 and https://www.rcsb.org/3d-view/2BEG/0, respectively (both links were accessed on 7 October 2023).

Although currently we observe a great interest and also encouraging results in the research on peripheral biochemical markers for AD [24], a definitive diagnosis was initially made only after histological analysis of the brain at autopsy. Positron emission tomography scanning technology (commonly indicated by the abbreviation ‘PET’) [25], which makes use of the C11-labeled Pittsburgh Compound B (abbreviated as ‘PiB’) [26], which is a thioflavin T derivative capable of selectively binding to amyloid-β (Aβ, Figure 1), generated sometimes-contradictory reports [20].

**Figure 2:**
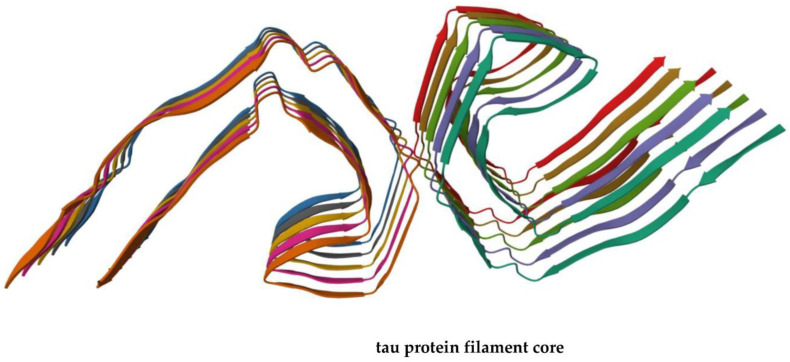
Three-dimensional view of a tau protein filament core made of two identical protofilaments comprising residues 306–378 of tau protein (PDB ID: 5O3L), whose aggregation is observed in AD. The reported structure is freely available at the link https://www.rcsb.org/3d-view/5O3L/1 (accessed on 7 October 2023).

The main problem is that by binding to amyloid beta, PiB does not always allow one to distinguish symptomatic from asymptomatic AD patients carrying amyloid plaques, the entities formed by self-assembly of Aβ fibrils (Figure 1). Moreover, the interaction of PiB with Aβ is influenced by the secondary and tertiary peptide structure of Aβ, which furnishes an explanation of the false-negative results that are observed in some cases. The progressive cognitive decline in older individuals with AD is accompanied by the occurrence in the brain of pathological aggregates not only made of Aβ but also of the phosphorylated form of tau protein [27,28] that is able to form filaments involved in neurodegeneration (Figure 2) [29].

Synaptic loss strongly correlates with cognitive decline in both animal models of AD and AD patients and, interestingly, soluble forms of Aβ and tau are believed to cause synaptotoxicity and spread through neural circuits, which results in neuronal loss and synapse degeneration and ultimately leads to brain atrophy [30].

## 3. DNA Vaccines against AD

**Figure 3:**
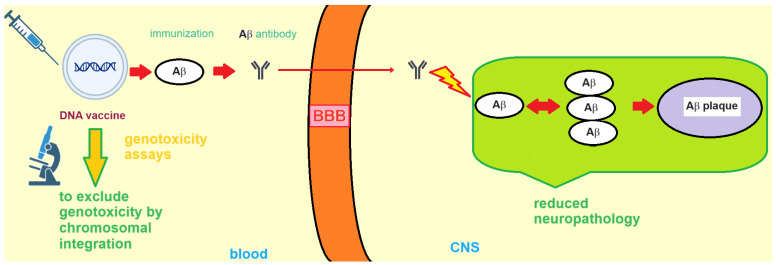
Schematic representation of the main idea behind Aβ-targeting DNA vaccination against AD. Specific Aβ antibodies target Aβ-peptides for clearance. BBB stands for blood–brain barrier; CNS stands for central nervous system.

In 1999, Schenk et al. first reported that Aβ deposition in murine models of AD could be reduced by active vaccination with Aβ peptide [17]. Clinical and preclinical data suggested that the development of an efficacious and safe anti-Aβ immunotherapy for Alzheimer disease requires therapeutic levels of anti-Aβ antibodies. At the same time, the immunization strategies should avoid autoreactive T cells and pro-inflammatory adjuvants, both of which are potentially able to increase the incidence of adverse events in the elderly patients targeted to receive the anti-Aβ immunotherapy. The first active immunization clinical trial used the AN1792 Aβ_1–42_ vaccine in AD patients, but its usage was subsequently halted because some of the individuals who received the AN1792 vaccine developed meningoencephalitis [31]. Another approach that was examined consisted in the passive immunotherapy, which made use of a monoclonal antibody designed to target the end terminus of the amyloid beta peptide. However, during the Phase II stage of the study the passive immunotherapy also showed adverse events that were dose-dependent, and consisted mainly in cases of vasogenic edema, as observed in 12 cases, with these adverse effects being particularly common in ApoE4 carriers, i.e., patients who are carriers of the ε4 allele that are particularly vulnerable to developing dementia. A possible remedy could consist in treating AD patients with lower doses of immunotherapeutics, particularly in the case of patients who are ApoE4 carriers. Since individuals in the prodromal or preclinical stages of Alzheimer disease pathogenesis cannot be easily identified, passive immunotherapy is reserved for those individuals that already show clinical AD symptoms, but sadly those same patients have already accumulated substantial neuropathology in the affected regions of their brains. Moreover, when tau pathology is found to be self-propagating and amyloid pathology itself drives tau pathology, which was observed in different animal AD models, then it may be crucial to administer the patients an early immunotherapy in the attempt of achieving positive clinical outcomes. That said, active immunization has several significant advantages, as the active immunization protocol is typically less intrusive to the AD patient and is also endowed with lower costs relative to passive therapy. In the case of adverse events induced by the Aβ-antibody immune complex, the patients need treatments with immuno-suppressants, with the treatments being administered for a suitable period of time, until the antibody levels in the patients decrease spontaneously as the consequence of the decays over time of the vaccine. Improving the design of the vaccines is a winning strategy aimed at improving both the safety profile and the effectiveness of the anti-amyloid immunotherapy. To overcome the issues encountered with amyloid vaccines, DNA vaccines were investigated as immunotherapy for AD (Figure 3).

The interest in this vaccine platform was reinforced by the observation that DNA vaccines are simple tools, easy to modify, and can be used without the need of adjuvants. Remarkably, DNA vaccines developed by several research groups were capable of reducing Aβ levels in AD mouse models without showing any important adverse effects, suggesting that DNA immunization may open the door to new prophylactic and therapeutic strategies against AD in the near future [17]. Interestingly, it was proven that the immunization with a DNA plasmid encoding Aβ alone (Figure 3) was able to determine an attenuated immune response that is sufficient to eliminate the amyloid pathology in animal models of AD [32]. Okura and Matsumoto developed non-viral DNA vaccines, administered these vaccines to APP23 mice, and eventually evaluated the resulting Aβ burden reduction in both therapeutic and prophylactic protocols. In particular, using their Aβ-Fc vaccine (Table 1) they achieved significant reductions in Aβ in the genetically-modified mice used as AD models without any significant side effects [33].

Overall, the above conclusions seem to corroborate the concept that DNA vaccines can disclose many favorable properties over the conventional approaches of passive or active vaccination against AD. In other terms, AD vaccination based on nucleic acids and especially DNA may lead to new scenarios for effective vaccine therapy against Alzheimer disease [34].

Since different kinetics are observable, as the immune response to DNA vaccination and the antibody production can also be low, a new vaccination scheme including two different prime-boost regimens was investigated. In particular, the two prime-boost regimens included an Aβ_1–42_ DNA prime plus an Aβ_1–42_ peptide boost, as well as an Aβ_1–42_ peptide prime plus an Aβ_1–42_ DNA boost. These were examined for their capability of eliciting antibody production and also for possible side effects connected with the consequent inflammatory response of T cells. While the two boost regimes significantly enhanced the production of specific antibodies, also showing similar antibody levels, no Aβ_1–42_ T cell response and, thus, no T cell proliferation or cytokine production were observed, which is in agreement with other studies that made use of an Aβ_1–42_ trimer DNA immunization and were able to substantially improve the safety of the process, which is of particular relevance for possible clinical use of this strategy [35]. Mechanistically, not only oligomers of Aβ peptide but also different amyloidogenic peptides as well as other amyloid beta species are endowed with neurotoxicity in AD, playing a key role in AD pathogenesis. In this regard, the YM3711 DNA vaccine (Table 1) acted as a powerful tool, targeting a wide range of AD-related species [36].

The DNA vaccination for anti-Aβ immunotherapy has the above-mentioned advantages, but also shows several hurdles, such as various infections, immunosenescence, and the need of selection of appropriate molecular adjuvants. A further refinement of DNA epitope vaccines for AD, also with the combined application of a prime-boost regime, is believed to make it possible to translate such a vaccination scheme to human clinical trials in either the preclinical AD stage, as diagnosed thanks to the use of effective AD biomarkers, or less preferably in very early AD [37].

The above-mentioned Aβ_1–42_ trimer DNA vaccine was suggested not only as a potential prophylactic tool for AD prevention but also as a way to slow down AD progression. AV-1959D (Table 1), a DNA vaccine directed against the epitope at the end terminus of the Aβ peptide, presented good immunogenicity in different animal AD models, including murine models of AD and, very importantly, non-human primates. After biosafety assessment, no significant adverse effects of the DNA immunization were observed in mice in both the short and long term. Additionally, mice treated with AV-1959D presented elevated levels of anti-Aβ antibodies that remained appreciable over time [38,39]. Recent studies showed that DNA vaccines targeting Aβ oligomers are able to ameliorate cognitive deficits of aged animal models of AD [40]. Moreover, a DNA-based epitope vaccine for AD (AV-1955, Table 1) was also found to be appropriate for human clinical testing, as revealed by a study in which humoral and cellular immune responses were evaluated in response to the administration of AV-1955 to *rhesus* macaques [41]. Nonetheless, DNA vaccination has historically encountered difficulties in the application to large mammals, with a main barrier to use DNA immunization in these animal models being the method employed for the delivery of the DNA vaccine [42]. Recently, a jet-injection modality was found to be effective at eliciting appreciable antibody production and safe immune responses in rabbits and mice, with no inflammatory cytokines being released after the treatment [42].

### 3.1. Formulation and Delivery of DNA-Based AD Vaccines

Since DNA vaccines are often not sufficiently immunogenic, a great attention is being currently paid to methods of engineering DNA-based vaccines, including prime/booster strategies, as well as the incorporation of adjuvants (co-delivered or transcribed/translated), all sharing the potential to enhance, prolong, or modulate antigen-specific immunogenicity of the DNA constructs [43].

As for the nature of the various adjuvants at disposal in DNA vaccines, whose appropriate choice is pivotal in optimizing the immunogenic response of the human body to a given vaccine, these are immunologic components able to raise the antigenic response of the nucleic acid-based vaccines and can be classified into two main categories: conventional and genetic. Conventional (or co-delivered) adjuvants are chemicals that can lead to stronger, more long-lasting, and optimized immune responses when administered with a vaccine. These can include mineral salts/gels; delta-inulin-based adjuvants; water-in-oil emulsions; oil-in-water emulsions; microbial derivatives; saponin-based adjuvants; cytokines and other endogenous human immunomodulators; cationic liposomes; virus-like particles; and combinations of the above. In particular, liposomes and virus-like particles are adjuvants often used by pharmaceutical companies to realize peptide vaccines for the treatment of Alzheimer disease [44]. On the other hand, genetic (transcribed or translated) adjuvants are nucleic expression vectors of different biomolecules, such as cytokines, that are capable of modulating the patient immune response after administration with the vaccine. Not less importantly, DNA itself possesses its own adjuvant activity without any need of external adjuvants, thanks to unmethylated CpG sites in specific regions that boost the innate immunity, acting as intrinsic immune adjuvants in DNA vaccines. Although they can be intrinsically immunogenic, the potency in vivo of DNA vaccines is often not sufficient in the case of humans and large animals, thus requiring different delivery methods, including electroporation or particle bombardment (also indicated as ‘gene gun’), to elicit good immunogenic responses in vaccinated subjects. In fact, a fundamental factor influencing the outcome of vaccination consists in the vaccine inoculation route. Famously, intramuscular vaccine injection predominantly leads to a Th1-type response, whereas a gene gun preferentially induces Th2-type immunity [45]. In more detail, this latter is a technique that delivers gold particles coated with DNA plasmids into the skin dermal and epidermal compartments and is believed to directly deliver DNA not only into the cells, but even into their nucleus, which explains the relatively low doses of DNA needed in this delivery system compared with others. It is noteworthy that Aβ-based DNA vaccines were administered with success by the gene gun route, which elicited appreciable humoral immunogenic responses in different animal models [45].

### 3.2. RNA Vaccines for Treatment of Alzheimer Disease

RNA vaccines have become a promising means to elicit immune responses as a reliable alternative to DNA vaccines, even though the latter ones are endowed with lower costs and more favorable manufacturing characteristics than RNAs. However, the efficacy of DNA vaccines in patients was found to be often insufficient, especially in terms of immunogenicity, and, on the other hand, the use of viral vectors often required for DNA vaccines was hampered by anti-vector immunity. In this context, RNA vaccines, such as self-amplifying RNA and mRNA vaccines, have the potential to avoid the drawbacks of DNA vaccines and viral vectors [46].

Despite the encouraging characteristics of RNA vaccines, to our knowledge there are very few studies so far that have investigated RNA vaccines for the therapy and prophylaxis of Alzheimer disease. The perspective article authored in 2021 by Prof. Jeffrey Fessel [47] supports the hypothesis that RNA vaccines and, particularly, mRNA self-replicating vaccines could increase the brain concentration of ATP by reducing Aβ levels, and consequently prevent the loss of cognition and future Alzheimer disease in elderly patients with synaptic hypometabolism. However, no experimental data were presented on the hypothesized mRNA self-replicating vaccine. Remarkably, among the research projects currently underway at the Monash University (Australia), it is worth mentioning that Prof. Colin Pouton and Dr. Rebecca Nisbet have started in July 2023 an experimental work aimed at developing a mRNA vaccine, in which the mRNA encapsulated within lipid nanoparticles ultimately targets tau protein, to be used for the therapy of Alzheimer disease. The expected end date for this research project is 31 December 2025 (https://research.monash.edu/en/projects/developing-an-mrna-vaccine-for-the-treatment-of-alzheimers-diseas: accessed on 2 November 2023). Interestingly, tau protein was the target of previously published works on active peptide vaccines for the treatment of Alzheimer disease, targeting either phosphorylated or non-phosphorylated tau protein, which also entered clinical studies [48]. Tau was the target also of a DNA vaccine developed using the universal vaccine platform technology MultiTEP that was able to induce high concentrations of anti-tau antibodies in murine models [49].

### 3.3. Nucleic Acid-Based AD Vaccines: Side Effects and Genosafety Profile

Owing to the side effects of Aβ DNA vaccines against Alzheimer disease, these need to be monitored with particular attention to evaluate, for example, the neuroinflammation and T cell proliferation induced by vaccination (Table 1) [33]. As for the genosafety profiles of RNA vaccines, enthusiasm for the ribonucleic vaccines has been motivated by the belief that their nucleic cargo is not expected to enter the cell nucleus or interact with the patient DNA, and thus RNA vaccines appear to have a safer profile than the DNA ones. For the latter vaccines, such a DNA integration event cannot be excluded a priori, but it would need to be assessed in human cell lines and vaccinated patients, especially in the case of adenoviral vaccines [50]. We are commonly subject to infections from DNA and RNA viruses that can lead to viral nucleic (DNA or RNA) acid inoculation into the human cells where integration events could, in principle, occur. Thus, one may argue that this natural event could be more probable than any potential nucleic acid integration from nucleic acid-based vaccines. Nevertheless, rigorous safety assessments of DNA and RNA vaccines should include genotoxicity studies that are clearly desirable to exclude that DNA and, after reverse transcription (demonstrated in eukaryotic cells), RNA vaccines could also drive oncogenic or other altered processes in human cells [51].

## 4. G-Quadruplex DNA and Alzheimer Disease: G4 Implication in AD and G4-Forming Sites in DNA Vaccines

**Figure 4:**
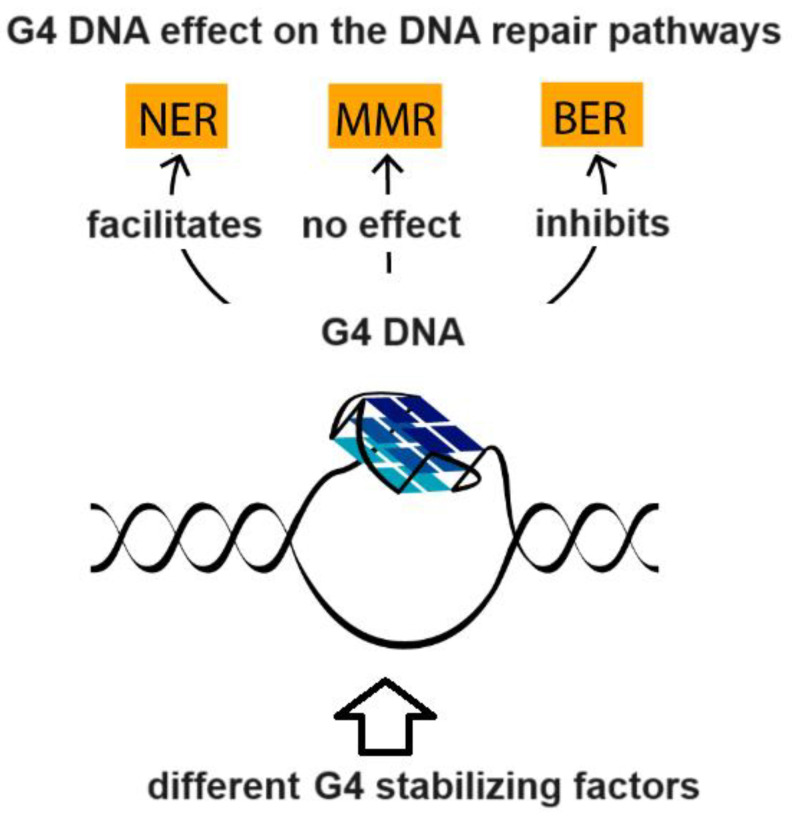
The effect of G4 DNA on DNA repair pathways. Note how G4 DNA stimulates the Nucleotide Excision Repair (NER) machinery of UV-induced DNA lesions, whereas they show opposite effects on the Base Excision Repair (BER)-mediated pathway. On the other hand, the interaction of the Mismatch Repair (MMR) proteins with the G4 structures does not correlate with any DNA repair activity (adapted from https://pub.mdpi-res.com/biomolecules/biomolecules-11-01284/article_deploy/html/images/biomolecules-11-01284-ag.png?1631066426: accessed on 31 October 2023).

G-quadruplex (G4)-forming oligonucleotides are higher-order RNA and DNA structures that recall a growing attention by the scientific community due to their implication in several disease states and biological processes occurring in many organisms [52,53,54]. Strategies aimed at manipulating G4 DNA, and some G4-driven biological processes, are leading strategies often explored in innovative studies in the attempt to discover new potential anticancer therapeutics or aptamers based on G4, to be employed in different biotechnological and biomedical applications [55,56]. Extremely crucial for cellular function, G4 DNA is implied in the regulation of a number of nucleic acid-based biomolecular processes, such as the replication, transcription, translation, and repair (Figure 4, [57]) of DNA, as well as the degradation of RNA.

Double-stranded DNA breaks at the origin of genomic instability are determined by the G4-forming DNA, as demonstrated by several studies, and G4-forming structures are also of pivotal importance in regulating biological events such as the formation of stress granules, which highlights the involvement of G4 structures in the aging process [58].

**Figure 5:**
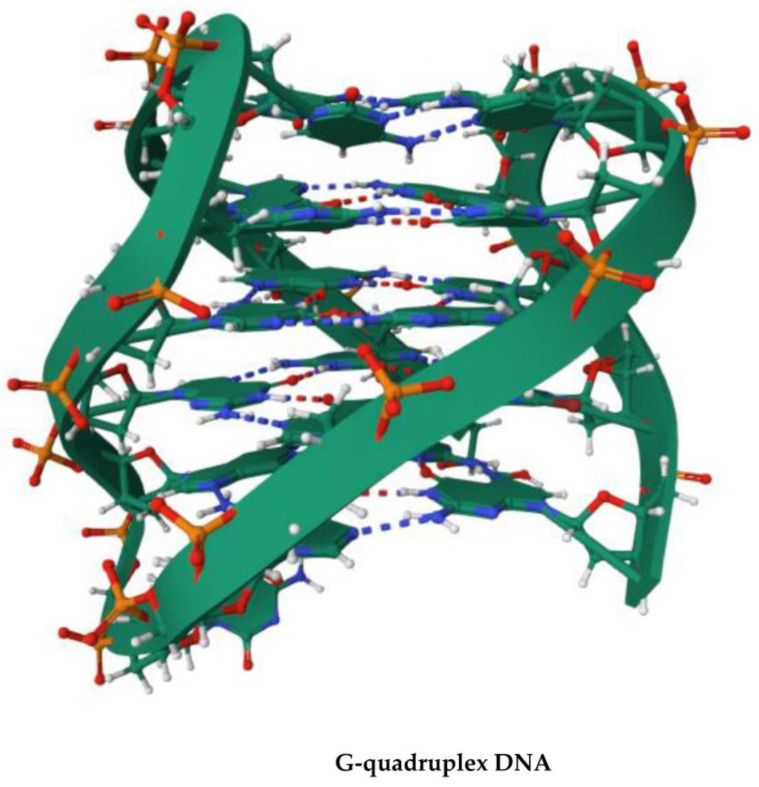
Three-dimensional view of the G4 structure of a DNA molecule containing d[GGGGCC]n repeats that is linked to ALS and FTD. The structure corresponds to the PDB ID: 5OPH and is freely available at the link https://www.rcsb.org/3d-view/5OPH/0 (accessed on 7 October 2023).

G4 structures of DNA sequences containing d[GGGGCC]_n_ repeats (see Figure 5) found in the non-coding tract of the gene C9orf72 are linked to neurological disorders like frontotemporal dementia (FTD) and amyotrophic lateral sclerosis (ALS) [59]. The role played by the G4 DNA helicases as well as their synergistic activity in the biology of aging and AD-related pathways raise optimism that effective targeting of G4 DNA could lead to new therapeutic scenarios following the discovery about how these nucleic structures promote aging in cell and neuropathy. The transition of the numerous G4-dependent biomolecular and cellular processes to the macroscopic aging and neuropathies such as AD is complex and may not be easily described. Many fundamental questions still wait an answer, such as how G4 DNA dynamics are regulated in the context of different cell types present in the brain. DNA repair and DNA damage responses may be associated differentially with amyloid beta and tau aggregation in AD, but which mechanism G4 and G4 helicases adopt to regulate these events remains unclear. A better comprehension of the implication of G4 DNA-dependent processes with molecular aging and neuropathological processes may help identify new therapeutic strategies and targets for future neurodrug discovery and development [58].

As already mentioned in this work, vaccination was proposed as one of the main strategies in the fight against COVID-19 [60]. The vast majority of vaccines used for this scope were based on DNA or mRNA molecules bearing genes encoding the viral Spike protein, which acted as an antigen for the human immune system. This approach seemed to be promising; however, some undesired effects (including symptoms classified from mild to severe) occurred in many individuals after the nucleic acid-based vaccination [60]. The explanation at a molecular level of the cause of such complications remains largely incomplete and unsatisfactory. In this respect, the G-quadruplex-forming potential in available DNA and RNA vaccines was proposed as one of the reasons [61]. Such G4-forming sites could potentially interfere with the expression of Spike protein in the case of COVID-19 vaccines or, more generally, any other antigens in the case of other vaccines, including the AD DNA vaccines. For example, the ChAdOx1 DNA vaccine was found to contain more than one hundred G4-forming sites, some of which had a very high probability of being formed in vivo and possibly interfering with different biomolecular processes. Thus, we propose that undesired G4-forming sites should be carefully checked as one of the first steps of new DNA and RNA vaccine development in case of Alzheimer disease and more generally in the context of any other disease prophylaxis and therapy. G4-forming sites can then be easily detected and removed from AD DNA vaccines using tools like G4Killer [62] or others.

## 5. Conclusions

In light of the knowledge currently available on DNA and RNA vaccines, we can state that these look to be promising tools for the treatment of Alzheimer disease. An evident advantage of nucleic acid-based vaccines is the easiness and higher rapidity of their manufacturing relative to traditional vaccines, as they can be immediately synthesized once the immunogen sequence is made available in the literature, and the productive process can be easily scalable. However, nucleic acid-based vaccines should undergo careful genotoxic assessment, as it cannot be excluded from consideration that the exogenous nucleic tracts introduced for the immunization could be integrated into the patient genome. Famously, researchers aim to provide effective treatments for medical conditions with minimal side effects. In recent decades, a number of studies on functional foods have been conducted to obtain foods with therapeutic activity [63]. Therefore, attempts to discover phytochemicals from food products with specific anti-AD properties are desirable as these could be part of a combined therapeutic approach using at the same time ‘food drugs’ and DNA and RNA vaccines. Ultimately, we believe that improving the currently known AD DNA epitope vaccines, to be used possibly in association with a prime-boost regimen, will facilitate the entry of AD DNA vaccines into human clinical trials involving patients in the early stages of AD, or, more desirably, individuals at a preclinical stage, as determinable by diagnosis using validated biomarkers of the Alzheimer disease.

## Figures and Tables

**Table 1:** Some characteristics of the main AD-related DNA vaccines discussed in this work.

Name	Animal Model	Strengths	Weaknesses
Aβ-Fc	APP23 mice	reduced Aβ burden no excessive neuroinflammation/T cell responses	no major weaknesses noted
YM3711	B6C3-Tg 85Dbo/J mice; New Zealand white rabbits; *cynomolgus* monkeys	significant reduction in Aβ and other amyloidogenic peptides in the brain	no major weaknesses noted
AV-1955	*rhesus* macaques	generates long-term and potent anti-Aβ antibodies	repeated (up to five times) immunization steps needed to achieve acceptable anti-Aβ antibody levels
AV-1959D	Tg2576 and Tg-SwDI mice	induces strong and therapeutically potent anti-Aβ antibodies with a favorable safety profile	low-grade reactions at the injection site

## Data Availability

Not applicable.
